# Indirectly acquired fear memories have distinct, sex-specific molecular signatures from directly acquired fear memories

**DOI:** 10.1371/journal.pone.0315564

**Published:** 2024-12-23

**Authors:** Shaghayegh Navabpour, Morgan B. Patrick, Nour A. Omar, Shannon E. Kincaid, Yeeun Bae, Jennifer Abraham, Jacobi McGrew, Madeline Musaus, W. Keith Ray, Richard F. Helm, Timothy J. Jarome

**Affiliations:** 1 Translational Biology, Medicine and Health Graduate Program, Virginia Polytechnic Institute and State University, Blacksburg, Virginia, United States of America; 2 Department of Pathology, Stanford University, Stanford, California, United States of America; 3 School of Neuroscience, Virginia Polytechnic Institute and State University, Blacksburg, Virginia, United States of America; 4 School of Animal Sciences, Virginia Polytechnic Institute and State University, Blacksburg, Virginia, United States of America; 5 Department of Biochemistry, Virginia Polytechnic Institute and State University, Blacksburg, Virginia, United States of America; Idaho State University, UNITED STATES OF AMERICA

## Abstract

Post-traumatic stress disorder (PTSD) is a severe anxiety disorder that affects women more than men. About 30% of patients suffering from PTSD develop the disorder by witnessing a traumatic event happen to someone else. However, as the focus has remained on those directly experiencing the traumatic event, whether indirectly acquired fear memories that underlie PTSD have the same molecular signature as those that are directly acquired remains unknown. Here, using a rodent indirect fear learning paradigm where one rat (observer) watches another rat (demonstrator) associate an auditory cue with foot shock, we found that fear can be indirectly acquired by both males and females regardless of the sex or novelty (familiarity) of the demonstrator animal. However, behaviorally, indirectly acquired fear responses resemble those of pseudoconditioning, a behavioral response that is thought to not represent learning. Despite this, using unbiased proteomics, we found that indirectly acquired fear memories have distinct protein degradation profiles in the amygdala and anterior cingulate cortex (ACC) relative to directly acquired fear memories and pseudoconditioning, which further differed significantly by sex. Additionally, *Egr2* and *c-fos* expression in the retrosplenial cortex of observer animals resembled that of demonstrator rats but was significantly different than that of pseudoconditioned rats. Together, these findings reveal that indirectly acquired fear memories have sex-specific molecular signatures that differ from those of directly acquired fear memories or pseudoconditioning. These data have important implications for understanding the neurobiology of indirectly acquired fear memories that may underlie bystander PTSD.

## Introduction

Post-traumatic stress disorder (PTSD) is a major anxiety disorder that affects women more than men [1, 2]. About 30% of individuals who witness a traumatic event, such as the victimization or death of another, may also develop PTSD [3–12]. This “bystander” PTSD has shown similar physiological and behavioral phenotypes to those who have experienced the trauma directly [13]. Although the underlying differences in their brain circuits and neurobiological signatures have not been well studied, both of these categories are diagnosed and treated the same way. Thus, a better understanding of the behavioral and neurobiological mechanisms that support the development of this bystander PTSD and how it differs from PTSD developed by individuals directly experiencing a traumatic event is needed to ensure the development of proper therapeutic interventions.

Since the 1980s, fear conditioning in animals has been proposed to recapitulate the main neurobiological aspects of PTSD and the fear circuits involved in its pathology [14, 15], making it a useful model for understanding the behavioral and biological mechanisms supporting the development of fear memories that underlie PTSD. From this model, we now know that new gene transcription and protein translation and degradation are critical in the amygdala to form a fear memory [16–18]. In terms of the latter, we have shown significant sex differences in the proteins that male and female rats target for protein degradation in the amygdala, suggesting a potential sex-specific role for ubiquitin-proteasome-mediated protein degradation in fear memory formation [19–21]. However, these fear conditioning models have largely focused on the individual experiencing the traumatic event. As a result, our knowledge of the molecular mechanisms involved in indirectly acquired fear memories remains limited.

To better understand the characteristics of indirectly acquired fear memories, scientists have developed an indirect fear learning (IFL) paradigm, also sometimes referred to as observational or social fear learning, with different species [22–26]. In rodents, indirect fear is acquired by observation of tone-foot shock pairing administered to a conspecific (demonstrator) [27–30]. Different factors, such as the role of the observer’s prior experience or the demonstrator’s vocalization or relationship to the observer, have been studied in the context of how fear memories can be acquired, though this has been largely done in male rodents [30–33]. Additionally, multiple brain regions have been demonstrated to be involved in IFL, such as the amygdala, similar to direct fear learning, and the anterior cingulate cortex (ACC) [13, 30, 32–35], partly overlapping with brain systems governing empathy [32, 36, 37]. Rodent and human studies have shown increased neural firing in the amygdala and ACC when experiencing their own pain and witnessing a conspecific receiving pain or signaling fear [27, 30, 32, 38, 39]. Moreover, the reciprocal connections between ACC and the amygdala are crucial for social fear transmission, creating vital questions about the nature of the amygdala and ACC involvement in IFL [40, 41]. However, to date, no prior study has examined if the molecular pathways involved in indirectly acquired fear are the same as those that are directly acquired in these brain regions.

In the present study, we studied the behavioral, molecular, and sex-specific mechanisms of indirectly acquired fear memories, focusing on the proteins targeted by degradation-specific K48-polyubiquitination as a readout of cellular memory consolidation. We found that both male and female rats can indirectly acquire fear in a sex- and novelty-independent manner. Additionally, the results suggest that even for a broad molecular mechanism such as protein degradation, there are distinct, unique molecular profiles when fear is acquired directly as compared to indirectly in both sexes. Importantly, while behaviorally indirect fear responses resemble those of pseudoconditioning, they are distinct at the molecular level. Collectively, these results reveal sex differences in the molecular mechanisms of indirectly acquired fear memories and that the molecular signature for memory formation might be different when fear memories are learned directly vs indirectly.

## Materials and methods

### Subjects

A total of 120 male and 104 female 8–9 weeks old Sprague-Dawley rats (Envigo, Fedrick, MA) were used for these experiments. All animals were housed two per cage, had free access to water and rat chow and were maintained under a 12:12-h light/dark cycle. Experiments and animal handling took place only during the light portion of the cycle. Animals were randomly assigned to experimental conditions. To ensure that the correct animal was used in the appropriate condition (i.e., observer, demonstrator, etc) personnel were not blinded to the experimental conditions during behavioral training. However, all behavioral testing data were scored by automated software to eliminate any bias. For molecular analyses, animals were given numerical identities that blinded personnel to the group assignments. All procedures were approved by the Virginia Polytechnic Institute and State University Institutional Animal Care and Use Committee (protocols #22–005) and conducted with the ethical guidelines of the National Institutes of Health.

### Fear conditioning apparatus

Two Habitest Chambers were used for fear conditioning and testing that were previously described [42] with several modifications. Each chamber consisted of a steel test cage with front and back Plexiglass walls and a grid shock floor above a plastic drop pan. The training chamber (context A) was separated in half with a wire mesh divider (1cm x 1cm holes), and the floor of one side was a stainless-steel grid; the other half was a black Plexiglas panel. The testing chamber’s (context B) walls were covered with orange and green tape to create a different context for the animals. A house light in the top back corner of the right wall of the chamber remained on during behavioral procedures. A USB camera was mounted on a steel panel outside the back Plexiglass wall of the chamber, angled at ∼45 degrees. The entire chamber was housed in an isolated cubicle with an acoustic liner and a house fan, which remained active during all behavioral procedures. Both chambers were equipped with a speaker centered in the middle of the right-side wall. Shock (during the training day) was delivered through the grid floor via a Precision Animal Shocker under the control of FreezeFrame 4 software, which also analyzed animal behavior in real time. A freezing threshold of 2.0 was used as the scoring parameter for all animals. All video was recorded and stored for later analysis. The chamber walls were wiped with 70% isopropanol (context A) or 4.25% of hydrogen peroxide (context B) before use.

### Indirect auditory-cued fear conditioning procedure

All animals were handled in the housing room for 2 days and then transported to an adjacent room where behavioral training was to occur and handled for 2 additional days. Auditory cued fear conditioning and fear testing were performed in two distinct behavioral chambers (context A and B), described above. Animals assigned to an experimental group requiring training (demonstrator, observer, or control) were then placed into the fear conditioning apparatus described above (context A). The demonstrator animals were placed in the side with stainless-steel grid floor, while observers and controls were placed into the other side of the chamber where the floor is covered with black plexiglass to prevent seeing the grid floor or receiving the shock stimulus. Following a 3 min baseline, demonstrators were exposed to six pairings of a tone (10 sec) co-terminating with a shock (1.0 mA, 1 sec) separated by 50-sec intervals, while the observer, on the other side of the mesh divider, was present during the conditioning procedure and heard the same tone presentation. The observer did not experience foot shock; however, they could see, hear, and smell the demonstrator rat through the mesh divider during the conditioning session. After a 1 min post-shock period, animals were returned to their home cage. Two days later, the control (did not have a demonstrator during training) and observer rats were separately placed into the same chamber (context A), the same side they were on the training session for five minutes, in the absence of a tone or a demonstrator to ensure the memory is not contextual. Next, the rats were transferred to context B (described above). After a 2 min baseline, animals were exposed to 12 unreinforced (no shock) tone (20 sec) presentations separated by 40-sec intervals and then returned to their home cage. All training sessions were recorded and scored live by FreezeFrame software. However, due to there being two animals in the training chamber all fear conditioning training sessions were hand scored using a continuous (every second) method. In all experiments, observer animals had no prior exposure to auditory cue, training context or shock before the indirect fear training. This was done to ensure that observer animals were naïve to these cues, allowing the formation of a new memory during training without the possibility of engaging reconsolidation or other memory updating processes.

### Tissue collection

One hour after the training session, experimental and naïve rats were overdosed on isoflurane in a necrosis chamber and the brain rapidly removed and immediately frozen on dry ice. Tissue containing the basolateral amygdala (BLA) and retrosplenial cortex (RSC) or the anterior cingulate cortex (ACC) were then dissected out by blocking the brain in a rat brain matrix (Harvard Apparatus, Holliston, MA) incubated with dry ice. All dissected tissue was frozen at -80°C and subsequently used for degradation-specific proteomics or qRT-PCR as described previously [20, 43, 44]. The same animals were used for the proteomic (Figs 5, 6) and gene expression (Fig 7) analyses, though only a subset was processed for proteomics and the RSC was lost from one cohort of females due to a freezer malfunction.

### Tandem Ubiquitin Binding Entity (TUBE) assays

BLA tissue was homogenized in buffer (10mM HEPES, 1.5mM MgCl2, 10mM KCl, 0.5mM DTT, 0.5% IGEPAL, 0.02% SDS, 70mM NEM, 1μl/ml protease inhibitor cocktail, and 1μl/ml phosphatase inhibitor cocktail) designed to preserve endogenous polyubiquitinated chains. Then a high affinity K48-polyubiquitin-selective tandem ubiquitin binding entity (K48-TUBE,100μl, #UM407M, Life Sensors, Malvern, PA) conjugated to beads was washed in buffer (100mM Tris-HCL, 150mM NaCl, 5mM EDTA, 0.08% NP-40) and a 500μl mixture of protein (300μg), protease inhibitor (1μg/ml), and Wash Buffer was added, followed by incubation for 2 hrs on rotator at 4°C. Samples were then washed twice and incubated at 96°C for 5 min at 800 rpm in 1X sample buffer (Bio-rad, Hercules, CA). After cooling at room temperature, the supernatant was collected and stored at -80°C for mass spectrometry analysis.

### Liquid Chromatography Mass Spectrometry (LC/MS)

Liquid chromatography mass spectrometry was performed as described previously [20]. The mass spectrometry proteomics data have been deposited to the ProteomeXchange Consortium via the PRIDE partner repository with the dataset identifier PXD041661 and 10.6019/PXD041661 (BLA) and PXD046274 and 10.6019/PXD046274 (ACC).

### RNA extraction, cDNA synthesis and quantitative real-time PCR

RNA was extracted with the RNeasy Universal Plus Midi-Kit (Qiagen, Germantown, MD). RNA concentration and quality were determined by Nanodrop. Normalized (200 ng), and converted to cDNA using the iScript cDNA synthesis kit (Bio-rad, Hercules, CA, USA). Real-time PCR amplifications of the cDNA were performed on the Bio-rad CFX96 Real-Time System using the following protocol: 95.0 ◦C for 3 min, then 95.0 ◦C for 10 s, followed by 60 ◦C for 30 s (39 repeats), 55–95 ◦C for 0.5 ◦C/cycle, followed by a melt curve starting at 55.0 ◦C for 10 s (81 repeats), and then held at 4.0 ◦C. Primers were: *Egr2* (F: AAGGCCGTAGACAAAATCCCA, R: CCAGCCACTCCGTTCATCTG), *c-fos* (F: CAGCCTTTCCTACTACCATTCC, R: ACAGATCTGCGCAAAAGTCC), and *Gapdh* (F: GGGCTGAGTTGGGATGGGGACT, R: ACCTTTGATGCTGGGGCTGGC) which was used as an internal control and the results were analyzed with the comparative Ct method.

### Statistical analysis

Outliers were defined as data points falling 2 standard deviations from the group mean using Prism software (GraphPad Software, La Jolla, CA). All data are presented as group averages with standard error of the mean. The behavioral data were analyzed using a repeated-measures two or three-way analysis of variance (ANOVA), using Tukey as *post-hoc* for multiple comparisons, and the gene expression data were analyzed with one-way ANOVA.

## Results

### Both males and females can indirectly acquire fear memories

**Fig 1:**
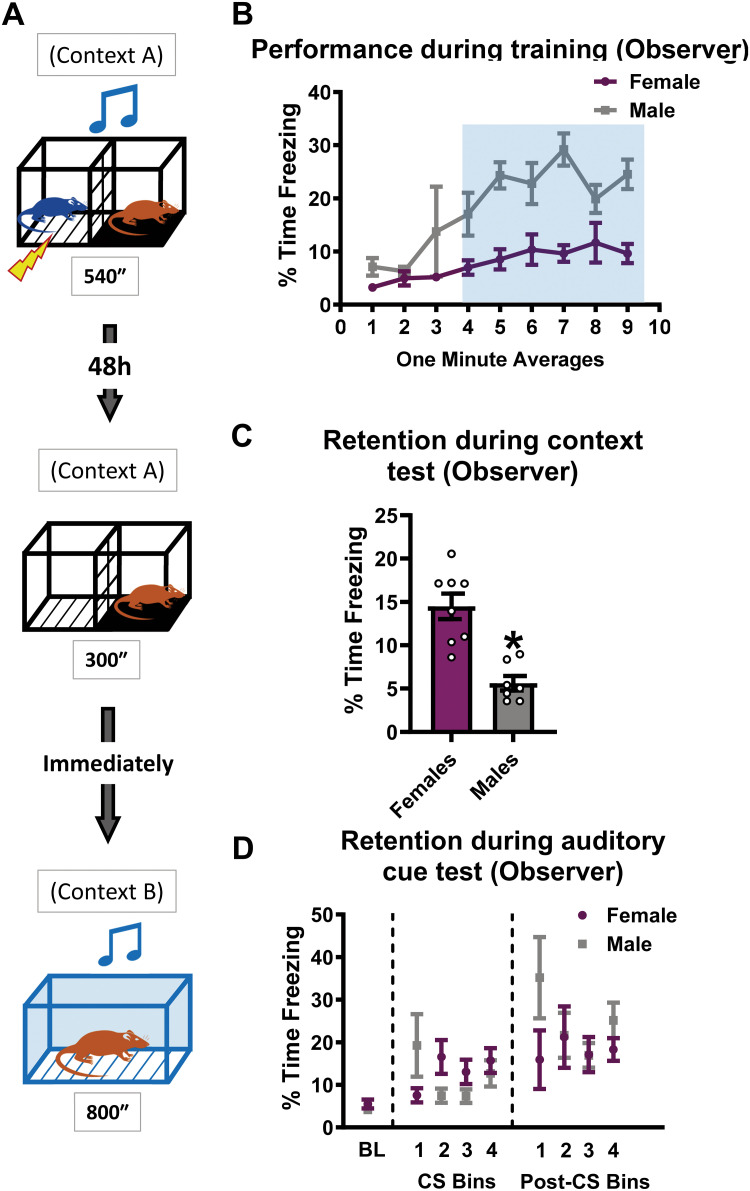
Males and females can both indirectly acquire fear memories. **(A)** Experimental design. The observer rats watch the same sex non-cage mate demonstrator rats undergo auditory fear conditioning in Context A. After two days, the observer is placed back into the training context for 5 minutes without receiving any foot shock or auditory cues and immediately transferred into Context B to be tested for fear to the auditory cue. **(B)** Behavioral performance of the observer during the training session. Both males and females show increased freezing after the first shock presentation to the demonstrator, though females showed less freezing behavior overall than males (minutes 4–9). **(C)** Behavioral performance of the observer during the training context retention test. **(D)** Memory retention of the observer during (CS) and between (post-CS) auditory cue presentations during the test session. Both sexes exhibited increased freezing behavior in response to the tone. Testing is shown as a bin of 3 CS or post-CS periods. N = 8 per group. Data show as mean with SEM. * Denotes p < 0.05 from females.

We used an indirect fear auditory conditioning paradigm to study sex differences in the behavioral and molecular mechanisms of directly vs indirectly acquired fear memories. In this model (Fig 1A), rats were trained with a conditioning chamber (context A) divided in half by a mesh divider, where a rat (observer) is put on one side with a covered floor by plexiglass, while another same sex non-cage mate rat (demonstrator) is placed on the other side where the floor is a stainless-steel grid through which foot shock is delivered following a brief auditory cue (tone) presentation that both animals can hear. Surprisingly, we found a significant effect of sex in performance of the observer animal during the training session. While female observers’ freezing behavior increased over time, it was significantly lower than that of male animals (Fig 1B, two-way repeated measure (RM) ANOVA, TIME: F_8,96_ = 5.715, *p* < 0.0001; SEX: F_1, 12_ = 51.16, *p* < 0.0001; TIME x SEX: F_8, 96_ = 1.769, *p* = 0.0926, N = 7 per group). This suggests that male and female observer animals differ dramatically in their overall fear responses during the training session. However, this could potentially be explained by female rats being more likely to display different behaviors, such as darting [45], during the training session, though such alternative behaviors were difficult for us to assess in the partitioned chamber.

After two days, the observer was placed back into the training chamber with the mesh divider to test contextual fear memory. During this test, we found that females froze significantly more than males (Fig 1C, unpaired *t*-test, *t* = 5.033, *p* = 0.0002 N = 8 per group), suggesting that there were sex differences in the level of contextual fear acquired during the indirect fear training. Immediately after this, the observer rat was placed into Context B for testing retention to the auditory cue. Both males and females exhibited increased freezing behavior in response to the tone conditioned stimulus (CS) both during (CS) and between (post-CS) auditory cue presentations with no significant effect for sex (Fig 1D; two-way RM ANOVA, CS: TIME: F_4,56_ = 2.910, *p* = 0.0294, SEX: F_1,14_ = 0.3854, *p* = 0.5447, TIME x SEX, F_4, 56_ = 3.287, *p* = 0.0172; Post-CS: TIME: F_4,56_ = 6.986, *p* = 0.0020, SEX: F_1,14_ = 1.011, *p* = 0.3318, TIME x SEX, F_4, 56_ = 2.002, *p* = 0.1068, N = 8 per group). These results indicate that male and female rats can indirectly acquire fear to an auditory cue from observing another rat undergo auditory fear conditioning.

### Neither novelty nor sex of the demonstrator control the indirect acquisition of fear memory

**Fig 2:**
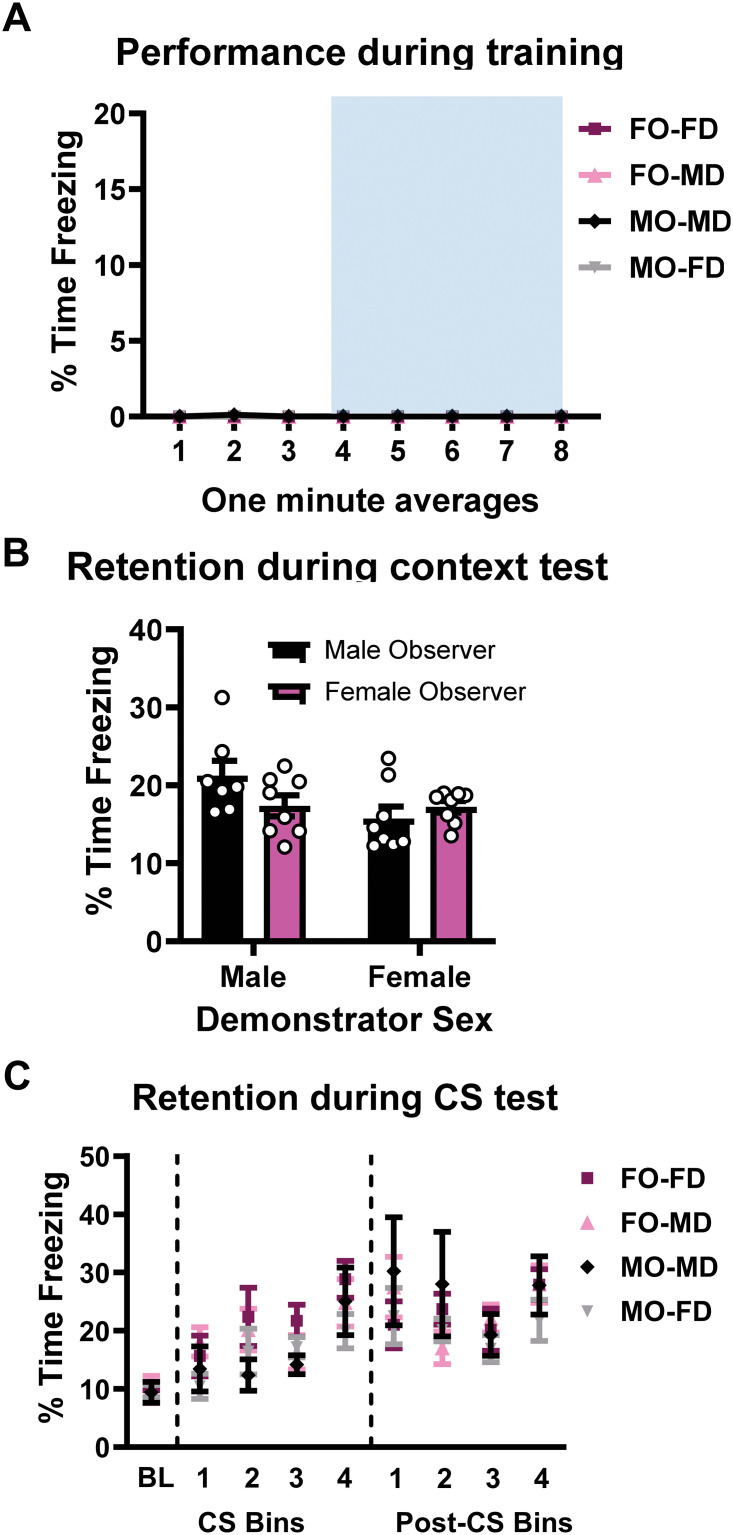
Sex of the demonstrator does not affect the acquisition of indirectly acquired fear memories. **(A)** Male and female observer rats were paired with a demonstrator of either the same or opposite sex. (**B-C**) Changing the sex of the demonstrator did not affect the observer’s performance in either sex for retention to the context **(B)** or auditory cue (**C**). Auditory cue testing is shown as a bin of 3 CS or post-CS periods. N = 7–8 per group. Data show as mean with SEM. F = female, M = male, O = observer, D = demonstrator.

To investigate whether the sex of the demonstrator affects the ability of the observer to indirectly acquire fear memory, we repeated the experiment above except with four groups that varied the sex of the demonstrator and observer: Female observer with female demonstrator (FO-FD), female observer with male demonstrator (FO-MD), male observer with male demonstrator (MO-MD), and male observer with female demonstrator (MO-FD). During training, we did not observe measurable freezing behavior from any of the observer animals regardless of condition or sex (Fig 2A). Instead, most animals spend their time interacting with the divider or moving about the chamber. For the context test, while we did not find any main effects there was a strong trend for an effect of the demonstrator’s sex (Fig 2B; two-way ANOVA, OBSERVER’S SEX: F_1,27_ = 0.6907, *p* = 0.4132; DEMONSTRATOR’S SEX: F_1,27_ = 3.914, *p* = 0.0582; OBSERVER’S SEX x DEMONSTRATOR’S SEX: F_1,27_ = 3.600, *p* = 0.0685; N = 7–8 per group). The freezing data from the auditory cue test shows that all groups had increased freezing levels compared to baseline both during the CS and post-CS periods. However, no significant differences in the observer rat performance was found when the sex of the demonstrator changed (Fig 2C; three-way RM ANOVA, CS: TIME: F_4,108_ = 17.64, *p* = 0.0001; DEMONSTRATOR’S SEX: F_1,27_ = 3.430, *p* = 0.0750; OBSERVER’S SEX x DEMONSTRATOR’S SEX: F_1,27_ = 0.1017, *p* = 0.7522; Post-CS: three-way ANOVA, Post-CS: TIME: F_4,104_ = 21.18, *p* < 0.0001; DEMONSTRATOR’S SEX: F_1,27_ = 0.6007, *p* = 0.4450; OBSERVER’S SEX x DEMONSTATOR’S SEX: F_1,27_ = 1.081, *p* = 0.3077, N = 7–8 per group). These data indicate that both male and female animals can indirectly acquire fear from either sex.

**Fig 3:**
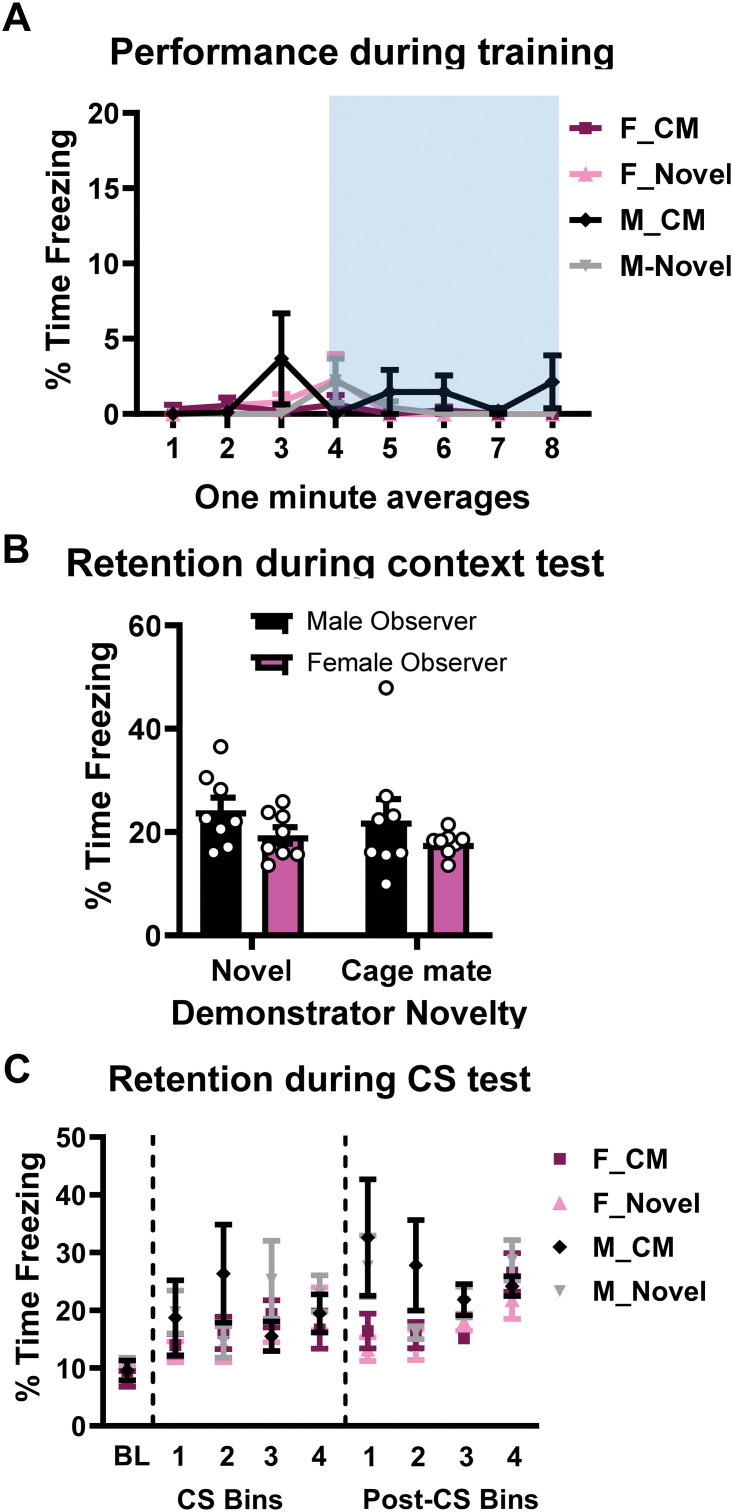
Novelty of the demonstrator does not affect the acquisition of indirectly acquired fear memories. **(A)** Male and female observer rats were paired with a demonstrator that was either a cage mate (familiar) or non-cage mate (novel) rat of the same sex. Changing the novelty of the demonstrator did not affect the observer’s performance in either sex during the testing session for the context (**B**) or auditory cue (**C**). Auditory cue testing is shown as a bin of 3 CS or post-CS periods. N = 8 per group. Data show as mean with SEM. F = female, M = male, CM = cage mate.

Next, we examined the role of demonstrator novelty on the ability to indirectly acquire fear in both males and females using four groups: Female observer with a cage mate demonstrator (F-CM), female observer with a novel demonstrator (F-Novel), male observer with a cage mate demonstrator (M-CM), male observer with a novel demonstrator (M-Novel). During training, we did not observe any significant differences in freezing behavior among sexes or condition (Fig 3A; three-way RM ANOVA, TIME: F_7,196_ = 1.316, *p* = 0.2446; OBSERVER’S SEX: F_1,28_ = 1.083, *p* = 0.3070; DEMONSTRATOR’S NOVELTY: F_1,28_ = 0.6423, *p* = 0.4296; OBSERVER’S SEX x DEMONSTRATOR’S NOVELTY: F_1,28_ = 1.944, *p* = 0.1742; N = 8 per group). For the context test, while we did not find any significant effects of the manipulation (Fig 3B; two-way ANOVA, OBSERVER’S SEX: F_1,28_ = 3.208, *p* = 0.0841; DEMONSTRATOR’S NOVELTY: F_1,28_ = 0.4533, *p* = 0.5063; OBSERVER’S SEX x DEMONSTRATOR’S NOVELTY: F_1,28_ = 0.0094, *p* = 0.9233; N = 8 per group). During the auditory cue test all groups exhibited increased freezing scores compared to baseline, however, there was not a main effect for demonstrator novelty (Fig 3C; three-way RM ANOVA, CS: TIME: F_2.499, 69.96_ = 5.835, *p* = 0.0024; DEMONSTRATOR NOVELTY: F_1, 28_ = 0.0002, *p* = 0.9886; OBSERVER SEX x DEMONSTRATOR NOVELTY: F_1, 28_ = 0.0714, *p* = 0.7913; SEX x DEMONSTRATOR NOVELTY x TIME: F_4, 112_ = 1.375, *p* = 0.2473; Post-CS: TIME: F_2.057, 57.60_ = 14.26, *p* < 0.0001; DEMONSTATOR NOVELTY: F_1, 28_ = 0.5692, *p* = 0.4569; OBSERVER SEX x DEMONSTRATOR NOVELTY: F_1, 28_ = 0.0703, *p* = 0.7928; OBSERVER SEX x DEMONSTATOR NOVELTY x TIME: F_4, 112_ = 1.139, *p* = 0.3419, N = 8 per group). These data indicate that the novelty of the demonstrator does not affect the performance of the observer of either sex.

### Pseudoconditioned rats show similar behaviors to observers

**Fig 4:**
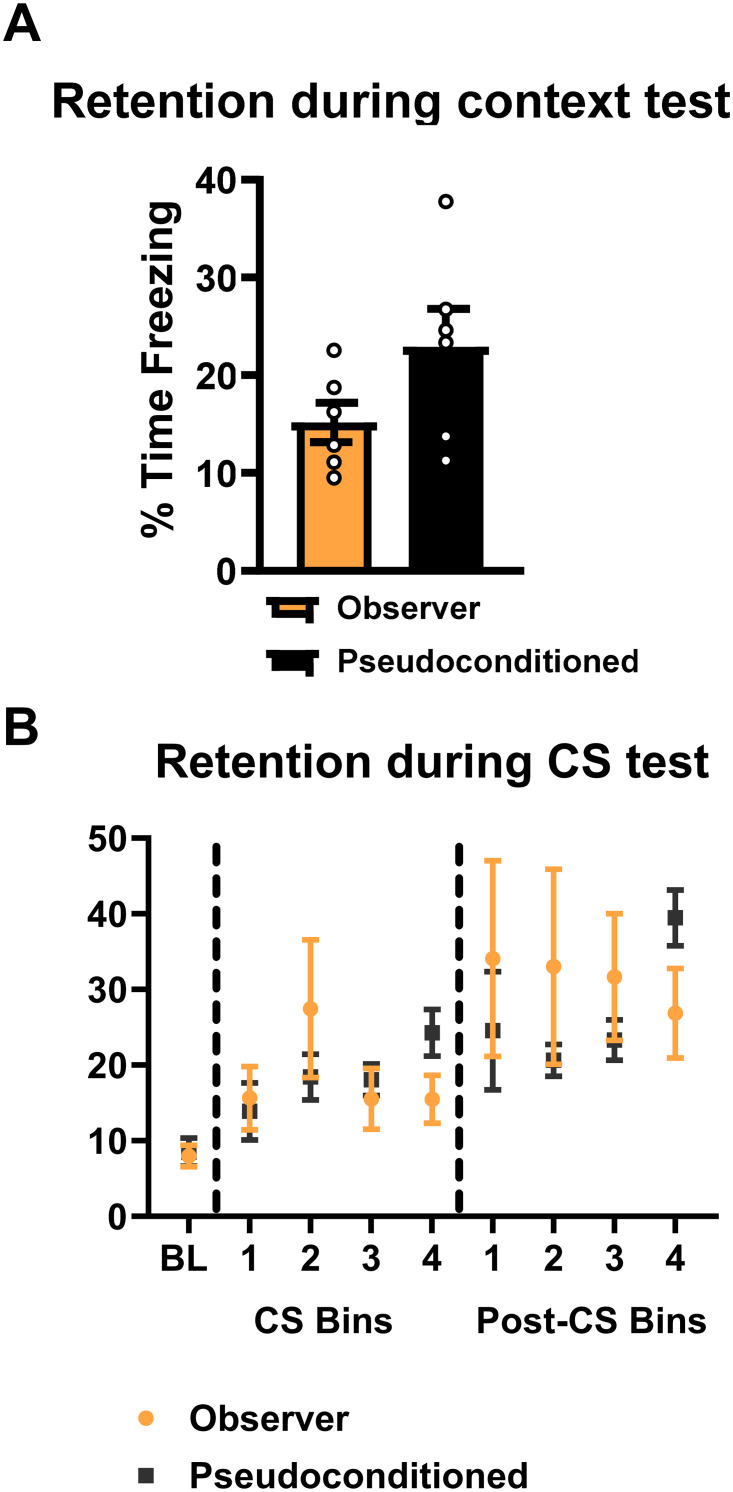
Indirectly acquired fear responses are behaviorally similar to those of pseudoconditioning. Rats were either trained with a novel same-sex demonstrator (observer) or without any demonstrators present in the partitioned chamber (pseudoconditioned) and then tested for the freezing response in context B two days later. (A-B) No significant differences were observed between the two groups during the context test (A) or during (CS) or between (post-CS) auditory cue presentations (B) during the test session. N = 6 per group, all males. Data show as mean with SEM.

Exposure to a novel auditory cue can result in increased freezing responses across time, even in the absence of shock, an effect called pseudoconditioning. As the auditory cue was the only variable shared among demonstrator and observer animals, we next tested if indirectly acquired fear responses are behaviorally similar to those of pseudoconditioning. Rats underwent training identical to that of the observers above except this occurred in the absence of a demonstrator animal. Similar to prior experiments, we did not observe measurable freezing behavior for observers with or without a demonstrator during the training session (*data not shown*). Further, during the context test we did not find a significant effect between groups (Fig 4A, unpaired *t*-test, *t* = 1.764, *p* = 0.1082, N = 6 per group, all males), though on average freezing behavior was higher in pseudoconditioned animals. Surprisingly, we did not find a significant difference between the observer and pseudoconditioned groups during CS and post-CS periods of the auditory cue test session (Fig 4B; Two-way RM ANOVA, CS: TIME: F_4,40_ = 3.928, *p* = 0.0088, GROUP: F_1,10_ = 0.0055, *p* = 0.9422, TIME x GROUP: F_4,40_ = 1.331, *p* = 0.2751; Post-CS: TIME: F_4,40_ = 4.325, *p* = 0.0053, GROUP: F_1,10_ = 0.3203, *p* = 0.5839, TIME x GROUP: F_4,40_ = 1.192, *p* = 0.3292, N = 6 per group, all males). These data suggest that at the behavioral level indirectly acquired fear responses are identical to that of pseudoconditioning.

### Indirectly acquired fear memories have unique, sex-specific protein degradation profiles in the amygdala relative to directly acquired fear memories and pseudoconditioning

**Fig 5:**
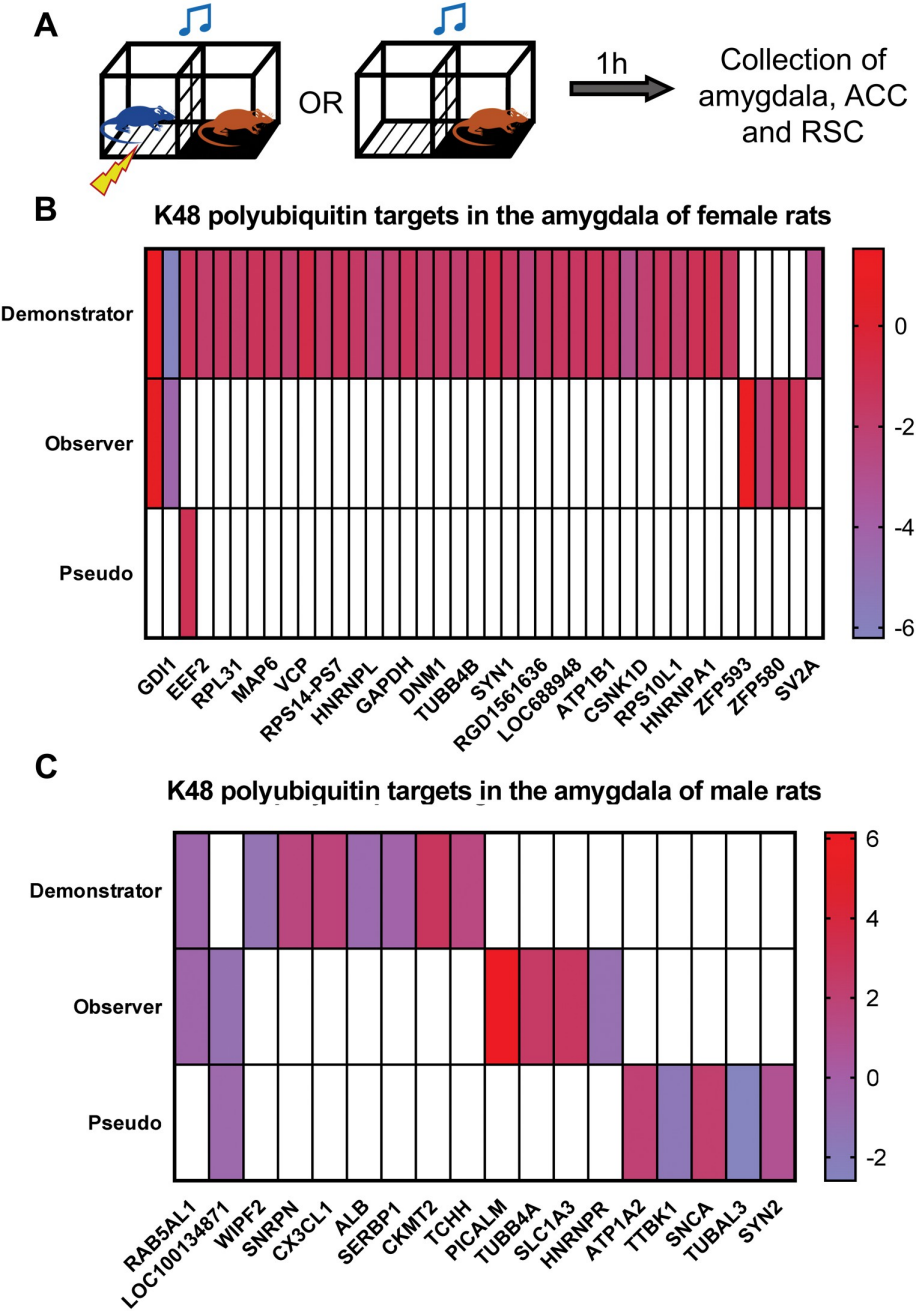
Indirect fear memories have unique, sex-specific protein degradation changes in the amygdala following training. (**A**) Male and female rats were trained in an indirect fear conditioning procedure that included a demonstrator (received tone and shock) and an observer (received tone while watching demonstrator). Pseudoconditioned animals were placed on the observer side of the chamber and presented with the tone without a demonstrator present. One hour after their respective training session, the basolateral amygdala (BLA) was collected, purified with K48-polyubiquitin Tandem Ubiquitin Binding Entity (TUBE) and analyzed with liquid chromatography mass spectrometry. All groups were compared to behaviorally naïve animals of the same sex with N = 6 per group per sex. (**B-C**) Heat maps showing the log2 value of proteins with significantly altered levels of K48-polyubiquitination (differentially ubiquitinated proteins; DUPs) in females **(B)** and males **(C)** in different training groups compared to naïve animals. White bars indicate that protein was not identified as significantly altered in that group.

We next tested if indirectly acquired fear memories have similar molecular profiles in the amygdala as those that are directly acquired and if this varies by sex. Further, as pseudoconditioning has been shown to have a unique molecular signature relative to direct fear learning [46], we assessed whether indirectly acquired fear responses were similar or different than that of pseudoconditioning. The ubiquitin-proteasome system (UPS) controls the majority of protein degradation in cells and is critical for fear memory formation. In this system, proteins that are “tagged” by lysine-48 (K48) polyubiquitin modifications will be degraded [47–49] and fear conditioning increases K48-polyubiquitination in the amygdala and other regions [16, 19, 42, 50, 51], a process that is dependent on the sex [19–21]. Thus, we focused on degradation-specific K48-polyubiquitination as a marker of the memory “consolidation” process. We trained male and female rats with indirect fear conditioning and collected the amygdala (basolateral; BLA) one hour later (Fig 5A), which has been reported as the peak time point for increased K48-polyubiquitination levels in the amygdala following fear conditioning [16, 21, 42]. For each sex, there were four groups: naïve, observer (an observer with a demonstrator), demonstrator, and pseudoconditioning (an observer without a demonstrator). K48-polyubiquitinated proteins were enriched by K48-TUBE purification followed by unbiased protein identification using LC/MS. In total, we found 657 proteins in females and 415 proteins in males that were targeted by K48-polyubiquitination. The higher number of proteins in females is consistent with our recent findings that females have naturally higher levels of protein degradation in the amygdala than males [21]. There were 40 and 18 proteins with significantly altered levels of K48-polyubiquitination (*P* < 0.05) in females (Fig 5B, **S1 Table in** S1 File) and males (Fig 5C, **S2 Table in** S1 File), respectively. Notably, these numbers represent only a small fraction of the total K48-polyubiquitinated proteins in each sex (Females = 6.24%, Males = 4.34%).

Next, we compared the differentially polyubiquitinated proteins (DUPs) in each trained group with the naïve rats in each sex and separated them by whether they had increased or decreased abundance in our TUBE-purified samples. We have previously shown that proteins with higher abundance (gain) in our enriched samples are going to be degraded, while those with lower abundance (loss) have actively undergone degradation [20]. In female demonstrators there were 36 DUPs, of which 35 had less abundance, and 1 protein, IQGAP3, a GTPase, had increased abundance in our samples following directly fear conditioning. In female observers there were only 4 proteins with less abundance and 2 with higher abundance. Interestingly, only 2 proteins, GDI1 (loss) and IQGAP3 (gain), overlapped with demonstrator animals, suggesting largely, but not entirely, different molecular pathways in the amygdala for direct and indirect fear learning in females. Additionally, the pseudoconditioned group only had 1 DUP, CAMKII (loss).

In contrast to females, male demonstrators had 8 while observers and pseudoconditioned each had 6 DUPs, of which half were more abundant in the pool of TUBE-purified samples compared to naïve rats in all groups. RAB5A, a small GTPase (gain), was found in both demonstrators and observers, and the only protein that overlapped between observers and the pseudoconditioned group was HBB1, a hemoglobin subunit. No proteins were found mutual between the sexes, indicating a sex difference in the fear-learning process that is consistent with our prior work. Taken together, our results show that even for a broad molecular mechanism such as protein degradation, the protein targets are largely unique to each condition and sex, suggesting that directly and indirectly acquired fear associations may have largely unique molecular signatures.

### Indirectly acquired fear memories have unique, sex-specific protein degradation profiles in the anterior cingulate cortex (ACC) relative to directly acquired fear memories and pseudoconditioning

**Fig 6:**
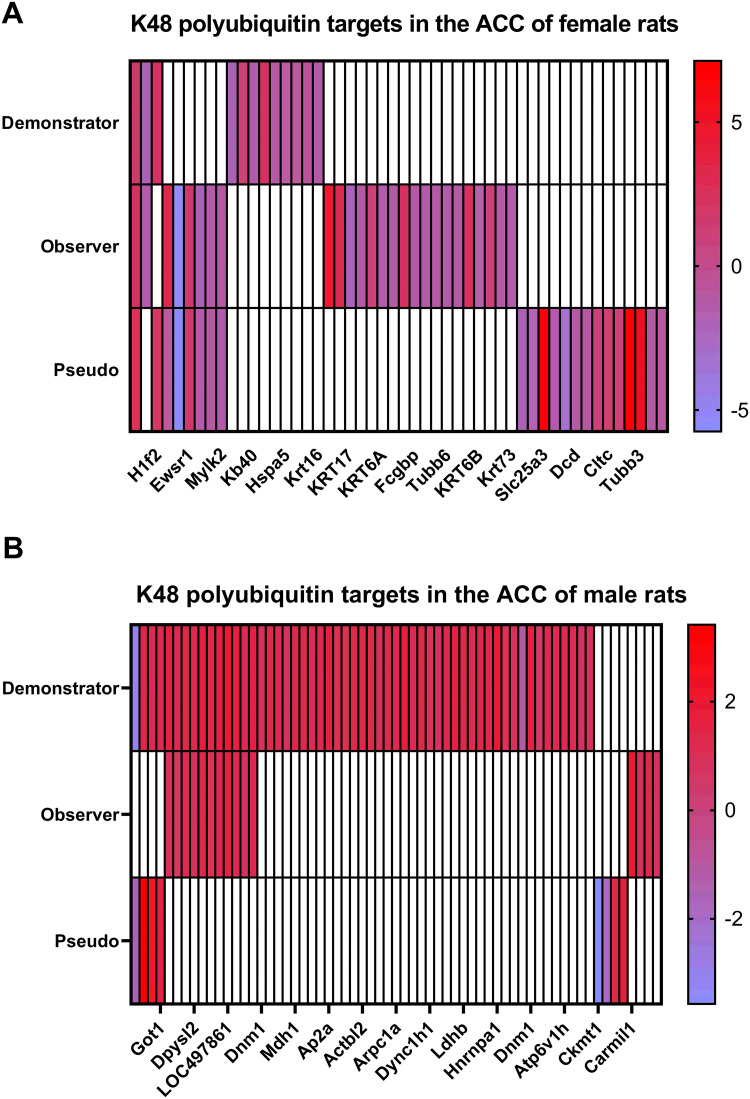
Indirect fear memories have unique, sex-specific protein degradation changes in the anterior cingulate cortex following training. Male and female rats were trained in an indirect fear conditioning procedure that included a demonstrator (received tone and shock) and an observer (received tone while watching demonstrator). Pseudoconditioned animals were placed on the observer side of the chamber and presented with the tone without a demonstrator present. One hour after their respective training session, the anterior cingulate cortex (ACC) was collected, purified with K48-polyubiquitin Tandem Ubiquitin Binding Entity (TUBE) and analyzed with liquid chromatography mass spectrometry. All groups were compared to behaviorally naïve animals of the same sex with N = 6 per group per sex. (**A-B**) Heat maps showing the log2 value of proteins with significantly altered levels of K48-polyubiquitination (differentially polyubiquitinated proteins; DUPs) in females **(A)** and males **(B)** in different groups compared to naïve animals. White bars indicate that protein was not identified as significantly altered in that group.

Next, we examined protein degradation profiles in the ACC, as there are numerous reports showing significant involvement of this area in indirect fear learning. In total, we found 481 proteins in females and 353 proteins in males that were targeted by K48-polyubiquitination. There were 50 and 63 proteins that showed significantly altered levels of K48-polyubiquitination (P < 0.05) in females (Fig 6A, **S3 Table in** S1 File) and males (Fig 6B, **S4 Table in** S1 File), respectively. The DUPs ratio to total K48-polyubiquitinated proteins is 10.39% in females and 17.84% in males, which are slightly higher than in the amygdala.

Next, we compared the abundance of DUPs in TUBE-purified samples of each trained group with the naïve animals separated by sex. In females, demonstrators had 12 DUPs, of which 8 had less abundance, while observers had 26 DUPs, with 9 proteins showing more abundance in our ACC samples following acquiring fear memories. Similar to the amygdala, only 2 proteins of the observers showed overlaps with the demonstrators, suggesting different molecular pathways are involved in the ACC region when fear is acquired directly or indirectly in females. However, unlike the amygdala, 22 proteins were differentially polyubiquitinated in the pseudoconditioned group, with 8 proteins overlapping with the observers and 2 with demonstrators.

In contrast to females, male demonstrators had 55DUPs, of which only 2 proteins had less abundance following the training, and observers had 15 DUPs (all gain), of which 11 proteins were also detected in the demonstrators (73.3% and 19.6% of the total DUPs detected in the observers and demonstrators, respectively). Interestingly, many of the DUPs from the demonstrator group were also identified in the amygdala samples of female demonstrators. Lastly, male pseudoconditioned animals had only 8 DUPs, half of which overlapped with demonstrators. There was no overlap between demonstrators or observers based on sex. Taken together, our results show that indirectly acquired fear memories have a molecular profile that is distinct from that of direct fear learning and pseudoconditioning, though overall, it more resembles the former than the latter.

### Indirectly acquired fear memories engage immediately early gene expression patterns in the retrosplenial cortex (RSC) that are similar to directly acquired fear memories but distinct from pseudoconditioning

**Fig 7:**
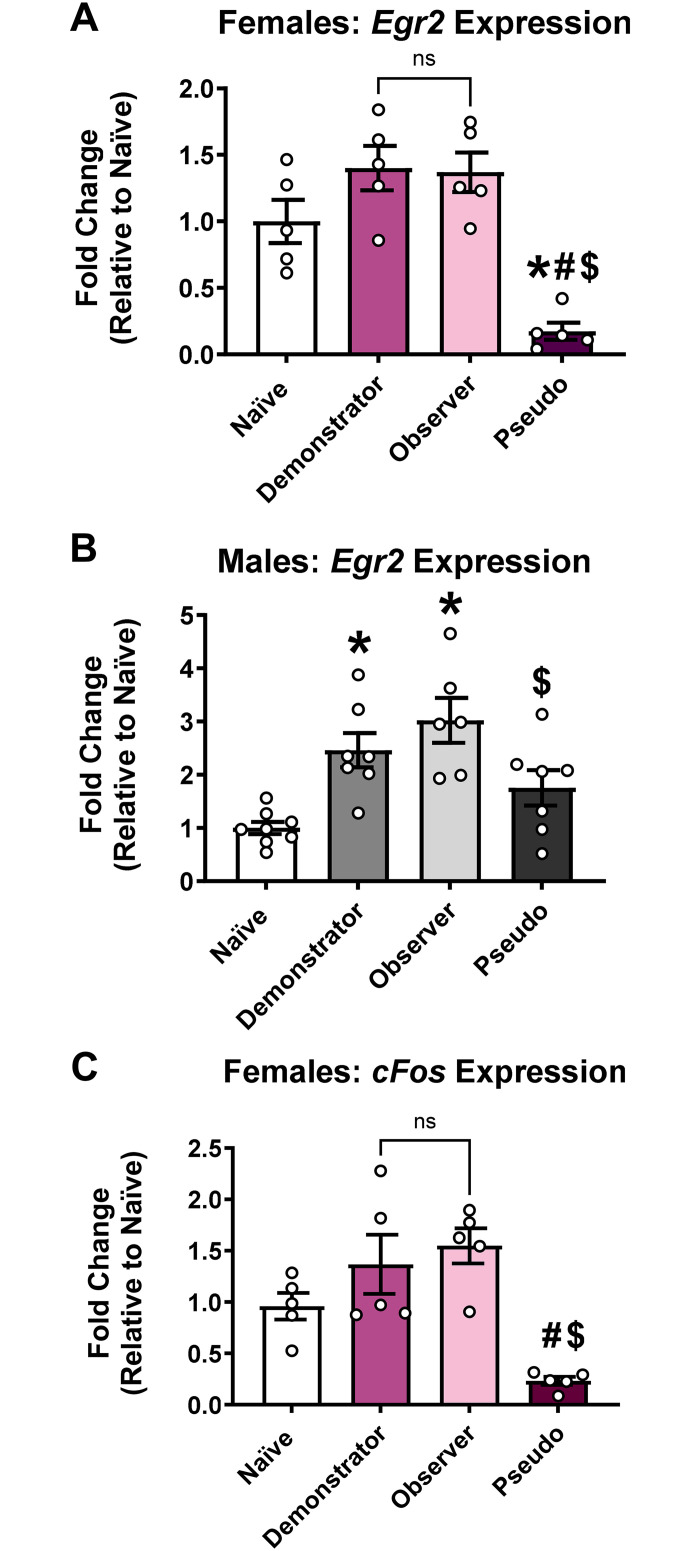
Indirectly acquired fear memories engage immediate early gene expression patterns in the retrosplenial cortex that are similar to directly acquired fear memories but distinct from pseudoconditioning. RNA was collected from the retrosplenial cortex from animals described above in Figs 4 and 5. Gene expression levels of two memory-related immediate early genes¸Egr2 and c-fos, were measured by RT-qPCR. (**A-B**) Egr2 mRNA levels were significantly higher in observer animals than pseudoconditioned animals in both females **(A)** and males **(B)**, though did not differ from demonstrator animals in either sex. (**C**) c-fos mRNA levels were significantly higher in observer animals than pseudoconditioned animals in females, though did not differ from demonstrator animals. N = 5 per group in females, and N = 6–8 per group in males. Data show as mean with SEM. NS: non-significant. * Denotes p < 0.05 from Naïve. # Denotes p < 0.05 from Demonstrator. $ Denotes p < 0.05 from Observer.

Next, we examined molecular signatures in the retrosplenial cortex (RSC) following indirect fear learning. While the RSC has yet to be studied in indirect fear learning, it is another cortical region involved in some forms of fear conditioning and is heavily connected to ACC [52, 53]. As we were unable to collect enough RSC protein to perform proteomic analyses, we instead examined gene expression levels of two memory-related immediate early genes, *Egr2* and *c-fos*. In both sexes (N = 5 per group in females, 6–8 per group in males), we found a main group effect for *Egr2* (Fig 7A and 7B; Females: F_(3, 16)_ = 16.33, *p* < 0.0001; Males: F_(3, 24)_ = 8.655, *p* = 0.0005). *Post-hoc* comparisons indicated that in both sexes observers have comparable levels of expression as the demonstrators, though only in male observers and demonstrators were *Egr2* levels higher than naïve animals. Interestingly, pseudoconditioned animals showed a significant reduction in *Egr2* expression in females, and not males, compared to all groups, a result consistent with prior studies where pseudoconditioning causes reductions in RNA concentration in some brain regions [46]. We also examined *c-fos* expression in females and found a similar pattern (Fig 7C, F_(3, 16)_ = 10.46, *p* = 0.0005) with observers and demonstrators had similar levels of expression and were both significantly higher than pseudoconditioned animals. In combination with our prior proteomic data, these findings show that indirect fear learning induces a molecular profile that is distinct from that of pseudoconditioning and partially, but not completely, resembles that of direct fear learning.

## Discussion

While decades of studies have examined the molecular mechanisms of directly acquired fear memories, very few have examined those mechanisms controlling the formation of memories for indirectly acquired fear associations. Using an indirect fear learning paradigm in rats, we found that both male and female rats can indirectly acquire fear in a sex-and novelty-independent manner Importantly, our data show that even for broad molecular mechanisms such as protein degradation or immediate early gene expression, the molecular signature of indirect fear memories in the amygdala and cortex (ACC) is largely unique relative to that of directly acquired fear memories in both sexes, though there are notable sex differences present in both of these regions. Importantly, our findings show that while behaviorally indirectly acquired fear responses are similar to those of pseudoconditioning, these are molecularly distinct processes. Together, these findings indicate that while indirectly acquired fear memories share some characteristics with that of directly acquired fear responses and pseudoconditioning, they are molecularly distinct from both.

Our data show that male and female rats can indirectly acquire fear to an auditory cue by observing another rat of either sex undergo auditory fear conditioning. While there are several examples where male rodents were shown to acquire fear indirectly or by proxy [28–30, 35, 54–56], there have been fewer studies on females [57, 58]. In the present study, we showed that either sex exhibited increased freezing during the test day, indicating they had learned the task indirectly through the demonstrator. Although females did not freeze as much as males during the training session in one of our experiments, this sex effect was not consistent across experiments and it has been suggested that females respond to trauma differently [59–61]. In fact, Gruene et al. [45] proposed that females respond to fear by darting behavior rather than freezing during the conditioning period, suggesting that both males and females do acquire the fear indirectly, however, at times they may express the fear in different ways.

Our study highlights new findings on the importance of demonstrator novelty by comparing observer performance when paired with a cage mate vs. a novel rat in both sexes. The results indicate that neither males or females react differently to the novelty of the demonstrator. Our findings from females confirms previous reports stating no effect for familiarity in females [62]. However, our finding in males is in contrast to others that have shown that the male observer response is positively influenced by being paired with a familiar demonstrator, such as siblings, long-term female mating partners, or genetic relatives [30, 33, 62]. However, these studies were conducted with mice or did not compare a cage mate to a novel, non-relative demonstrator, which could explain the different observations.

One surprising finding was that the behavioral performance of pseudoconditioned animals during the testing session closely resembled that of observers, suggesting that indirectly acquired fear associations may be a product of pseudoconditioning as opposed to associative learning. Notably, prior studies have rarely used the observer alone control group that we did in the present study. However, while behaviorally indirect fear learning and pseudoconditioning are similar, our data from the amygdala, ACC and RSC show a clear distinction of these two paradigms at the molecular level. Thus, while indirectly learned auditory fear associations are indistinguishable from pseudoconditioning at the behavioral, they have molecularly distinct processes, suggesting that indirect fear memories are not a product of pseudoconditioning.

Another interesting finding from our study was that the RSC was engaged during the formation of both direct and indirect fear memories. The RSC is strategically situated for facilitating information exchange among higher-order brain regions, given its direct reciprocal connections with the prefrontal cortex, ACC, and hippocampus [63, 64]. Notably, the RSC is critically involved in fear conditioning [65, 66] and the memory permissive genes, *Egr2* and *c-fos*, are robustly induced following training in this region [67]. Gene expression data from *Egr2* and *c-fos* in females in our study align with the previous findings of Knapska et al. reporting similar level of *c-fos* activation in observers and demonstrators in the amygdala [34]. The comparable induction of *Egr2* in both sexes, in both the direct and indirect learning groups, indicates that the RSC may be equally involved in both types of learning. While this might be surprising considering that the RSC has not been traditionally considered to be involved in auditory (delay) fear conditioning [53, 63–65], at least at recent time points, it should be noted that the RSC does have a role in context fear conditioning [64]. As context is typically learned as a background cue in our direct auditory fear conditioning procedure and background context fear conditioning is impaired when protein synthesis is blocked in the RSC around the time of training [16, 64], it is likely that the engagement of RSC in our direct fear conditioning animals is contextually-driven. Moreover, the lower levels of expression observed in pseudoconditioned animals compared to observers imply that despite similar performances in behavioral analyses, observers are acquiring fear, similar to demonstrators, while the pseudoconditioned group is not, as discussed above. As our observer animals did show evidence of contextual fear conditioning (Fig 1C), it is possible that the RSC involved in both direct and indirectly fear conditioned animals is contextually driven. Regardless, the clear distinction between gene expression in the pseudoconditioned and observer animals indicates that indirect fear learning cannot be directly equated with pseudoconditioning.

To date, no prior study has examined whether the molecular mechanisms required for memory formation vary between directly and indirectly acquired fear in different brain regions. For the first time, we investigated this great gap in our knowledge by studying a broad molecular mechanism heavily involved in fear memory formation, protein degradation, using an unbiased proteomic approach. Our analysis of the protein targets for degradation in the amygdala and ACC revealed a) a clear distinction between trained vs pseudoconditioned animals, b) a largely unique molecular signature for direct vs indirect training conditions in females, and c) these signatures in males depend on the brain region. This suggests that there may be differences in the molecular requirements to form memories for directly or indirectly acquired fear associations. Of particular interest are the potential pathways that may be selectively impacted by protein degradation during the formation of directly acquired or indirectly acquired fear memories. For example, in the amygdala of females there were selective targeting of zinc finger proteins and, surprisingly, a major proteasome subunit (PSMC5) that recently has been shown to have a transcriptional co-factor role during fear memory formation [68]. Conversely, the protein degradation profile was broad in female demonstrator animals, targeting a variety of structural, ribosomal and intracellular signaling proteins. Interestingly, CaMKIIα was a target of protein degradation in female demonstrators and pseudoconditioned animals, but not observers, suggesting a unique overlap with directly acquired fear memories in pseudoconditioning. In the male amygdala the targets of protein degradation varied widely in function in all three conditions but notably diverged from that of females as there was little overlap between sexes. This latter result is consistent with our recent work demonstrating that male and female rats target different proteins with both degradation-specific and degradation-independent ubiquitin modifications in the amygdala during the formation of a contextual fear memory [20, 43, 44]. While it is unclear why males and females show such little overlap in target proteins in our analyses, our prior work suggests that this may be driven by resting level differences in the regulation and use of ubiquitin signaling mechanisms in the young adult amygdala [21, 69].

In the ACC, female demonstrators targeted a variety of proteins including those that are structural or chaperones while female observer rats primarily targeted structural proteins. Again, there was very little overlap with the protein degradation targets with males though these demonstrators targeted structural proteins and those involved in ATP synthesis, intracellular signaling and, surprisingly, ubiquitin while observers were mostly structural and those involved in ATP synthesis. Of particular importance is that the protein degradation profile for directly acquired fear memories was more robust in the amygdala of females but the ACC of males, suggesting interesting and unexpected sex-specific circuit differences in the molecular signature of directly acquired fear memories. Conversely, the robustness (number of targets) of the protein degradation profiles in observer animals was similar between sexes in the amygdala but slightly greater in females in the ACC. While it is unclear what the functional significance is for these sex differences in the scope of the protein degradation profiles across brain regions in a sex-specific manner, these data could have important implications for understanding the sex bias that exists in PTSD prevalence.

The findings presented here provide novel insight into the behavioral characteristics and protein degradation involved in indirectly learned fear individuals. However, there are some limitations to be acknowledged in the present study. One limitation is when investigating the K48-polyubiquitin mark for protein degradation, we did not study other degradation marks or time points. Future studies should use the same approach to examine how the protein degradation targets change across time during the formation of indirectly acquired fear memories. Moreover, we did not examine the transcriptome signatures of directly and indirectly acquired fear memories. Strong evidence demonstrates that gene transcription is essential for memory formation and that the transcriptome of the cells changes for different types of memories and brain regions [70, 71]. While the transcription signature of directly acquired fear memories have been studied before [72, 73], no studies have investigated the transcriptome profiles of indirectly acquired fear memories. However, the same could also be said for essentially any other molecular mechanism as the cellular mechanisms of indirectly acquired fear memories remains poorly studied. This will be an area of emphasis for future studies. It should also be noted that while we did not find an effect of animal familiarity/novelty on the ability to indirectly acquire fear memories, this experiment was done with animals delivered from a vendor and not bred in-house. As a result, we cannot exclude potential prior interactions non-cage mates may have had during shipment to our facility or at their prior facility, nor do we know if some may have come from the same litter. Future studies will aim to test the influence of animal novelty on indirect fear learning using animals bred in-house to better control for these additional variables.

Finally, while our data strongly suggest that indirect fear memories require a molecular signature that is largely unique from that of directly acquired fear memories or pseudoconditioning, it is important to note that due to the complex nature of our experimental design we were unable to assess also possible conditions in the present study. For example, we did not include a condition in which the observer animal received auditory cue presentations with a demonstrator present that does not receive any shock presentations, which would essentially induce pseudoconditioning in both the observer and demonstrator. However, as such a control still allows social interaction and the pseudoconditioned demonstrator would begin to show freezing (fear) responses, this could result in a learned fear response in observer animals. This is likely to engage the same molecular changes we observed in our present study, though this has never been directly tested. Further, our observer (and demonstrator) animals all displayed fear to the training context during test, suggesting that the hippocampus is likely engaged. It would be interesting to test if the molecular signature induced in the hippocampus is the same in observer and demonstrator animals, especially since pseudoconditioned rats tended to show the highest level of context fear. Additionally, we did not include a condition in which both the observer and demonstrator are in the training chamber together but do not receive any auditory cue presentation, which would allow social interaction but without both pseudoconditioning and presentation of the unconditioned shock stimulus. Such a scenario could potentially reveal important insights into the role of the RSC in indirect fear memories vs that of pseudoconditioning. Finally, it is unclear how prior exposure to the unconditioned shock stimulus, the training context or the auditory cue by the observer animals may have changed the molecular signature of the indirectly acquired fear memory, particularly since prior exposure could potentially result in a latent memory that might become updated during the training session. While out of the scope of the present study, future work will focus on better identifying the exact conditions that induce the specific molecular signature induced by indirect fear learning in a distributed network that includes the hippocampus, amygdala, ACC and RSC.

## Conclusion

In conclusion, we provide evidence that indirectly acquired fear memories do not engage the same molecular profile in the brain as memories for fear associations that are directly acquired. Further, we also found that these molecular profiles vary by sex, though males and females can equally acquire fear indirectly in a sex- and novelty-independent manner. These results have important implications for translational studies focusing on patients who develop PTSD indirectly.

## Supporting information

S1 File Supplementary tables. S1-S4 Tables.
(PDF)

S2 File Original data. Original data used to make graphs presented in Figs 1–4 and 7.
(XLSX)
