# Supplementary material for: Indirectly acquired fear memories have distinct, sex-specific molecular signatures from directly acquired fear memories
Source: PLoS One. 2024 Dec 23;19(12):e0315564. doi: 10.1371/journal.pone.0315564 (PMC11666066; doi:10.1371/journal.pone.0315564)
Supplement: S1 File — S1-S4 Tables. (PDF) [file pone.0315564.s001.pdf]

**SUPPLEMENTAL TABLES**

# Indirectly acquired fear memories have distinct, sex-specific molecular signatures from directly acquired fear memories

Shaghayegh Navabpour, Morgan B. Patrick, Nour A. Omar, Shannon E. Kincaid, Jennifer Abraham, Jacobi McGrew, Madeline Musaus, W. Keith Ray, Richard F. Helm, and Timothy J. Jarome

**Supplemental Table 1: K48 polyubiquitin targets in the amygdala of female rats**

| **Group** | **Protein** | **log 2 FC** | ***p*-value** |
|---|---|---|---|
| Demonstrator | EEF2 | -1.859 | 0.001 |
| Demonstrator | GDI1 | -6.208 | 0.003 |
| Demonstrator | YWHAB | -1.601 | 0.009 |
| Demonstrator | RPL31 | -1.888 | 0.012 |
| Demonstrator | GLUL | -1.333 | 0.013 |
| Demonstrator | MAP6 | -1.501 | 0.014 |
| Demonstrator | EEF1G | -2.016 | 0.017 |
| Demonstrator | VCP | -0.773 | 0.018 |
| Demonstrator | CADPS | -1.989 | 0.019 |
| Demonstrator | RPS14-PS7 | -1.399 | 0.019 |
| Demonstrator | SYNGR3 | -1.669 | 0.021 |
| Demonstrator | HNRNPL | -2.659 | 0.021 |
| Demonstrator | SPIRE2 | -2.026 | 0.026 |
| Demonstrator | GAPDH | -1.369 | 0.028 |
| Demonstrator | RPL26 | -1.986 | 0.030 |
| Demonstrator | DNM1 | -1.689 | 0.032 |
| Demonstrator | IQGAP3 | 1.528 | 0.032 |
| Demonstrator | AP2A2 | -2.010 | 0.032 |
| Demonstrator | SV2A | -2.874 | 0.033 |
| Demonstrator | TUBB4B | -1.352 | 0.033 |
| Demonstrator | HNRNPA3 | -0.925 | 0.034 |

| | | | |
|---|---|---|---|
| Demonstrator | SYN1 | -1.596 | 0.034 |
| Demonstrator | DYNC1H1 | -2.456 | 0.036 |
| Demonstrator | RGD1561636 | -1.611 | 0.036 |
| Demonstrator | FSCN1 | -1.462 | 0.037 |
| Demonstrator | LOC688948 | -1.529 | 0.039 |
| Demonstrator | TUBA1A | -1.288 | 0.040 |
| Demonstrator | ATP1B1 | -1.486 | 0.040 |
| Demonstrator | CRIP2 | -3.029 | 0.040 |
| Demonstrator | CSNK1D | -2.112 | 0.042 |
| Demonstrator | TUBB2A | -1.418 | 0.042 |
| Demonstrator | RPS10L1 | -1.939 | 0.045 |
| Demonstrator | CAMK2A | -1.258 | 0.046 |
| Demonstrator | LOC687780 | -1.199 | 0.048 |
| Demonstrator | HNRNPA1 | -0.952 | 0.049 |
| Demonstrator | RPS20 | -1.565 | 0.049 |
| Observer | GDI1 | -4.362 | 0.004 |
| Observer | ZFP593 | 1.304 | 0.014 |
| Observer | IQGAP3 | 0.733 | 0.021 |
| Observer | KRT77 | -2.438 | 0.027 |
| Observer | ZFP580 | -1.275 | 0.029 |
| Observer | PSMC5 | -1.231 | 0.030 |
| Pseudo | CAMK2A | -1.168 | 0.048 |

**Supplemental Table 2: K48 polyubiquitin targets in the amygdala of male rats**

| **Group** | **Protein** | **log 2 FC** | ***p*-value** |
|---|---|---|---|
| Demonstrator | RAB5AL1 | -0.493 | 0.006 |
| Demonstrator | WIPF2 | -1.290 | 0.011 |
| Demonstrator | SNRPN | 1.592 | 0.013 |
| Demonstrator | CX3CL1 | 1.783 | 0.036 |
| Demonstrator | ALB | -0.766 | 0.036 |
| Demonstrator | SERBP1 | -0.321 | 0.038 |
| Demonstrator | CKMT2 | 2.852 | 0.041 |
| Demonstrator | TCHH | 1.487 | 0.047 |
| Observer | LOC100134871 | -1.145 | 0.015 |
| Observer | RAB5AL1 | -0.467 | 0.015 |
| Observer | PICALM | 6.147 | 0.041 |
| Observer | TUBB4A | 2.514 | 0.047 |
| Observer | SLC1A3 | 2.755 | 0.048 |
| Observer | HNRNPR | -1.112 | 0.048 |
| Pseudo | ATP1A2 | 2.031 | 0.001 |
| Pseudo | TTBK1 | -1.660 | 0.025 |
| Pseudo | SNCA | 2.173 | 0.025 |
| Pseudo | TUBAL3 | -2.590 | 0.032 |
| Pseudo | SYN2 | 0.851 | 0.037 |
| Pseudo | LOC100134871 | -0.670 | 0.048 |

**Supplemental Table 3: K48 polyubiquitin targets in the ACC of female rats**

| **Group** | **Protein** | **log 2 FC** | ***p*-value** |
|---|---|---|---|
| Demonstrator | Rplp2 | -2.112 | 0.000 |
| Demonstrator | Aldoc | -2.132 | 0.002 |
| Demonstrator | Prr36 | 1.210 | 0.010 |
| Demonstrator | H1f2 | 2.072 | 0.019 |
| Demonstrator | Kb40 | -1.422 | 0.020 |
| Demonstrator | Chtop | 2.267 | 0.028 |
| Demonstrator | Hal | -1.120 | 0.034 |
| Demonstrator | Hspa5 | -0.975 | 0.034 |
| Demonstrator | Cat | -0.918 | 0.046 |
| Demonstrator | Evpl | 1.670 | 0.048 |
| Demonstrator | Ncdn | -0.698 | 0.049 |
| Demonstrator | Krt16 | -1.166 | 0.050 |
| Observer | LOC100361067 | 3.375 | 0.000 |
| Observer | Ercc3 | 4.587 | 0.000 |
| Observer | Sfpq | 2.781 | 0.001 |
| Observer | Ewsr1 | 1.599 | 0.006 |
| Observer | Aldoc | -1.591 | 0.006 |
| Observer | KRT17 | -2.178 | 0.006 |
| Observer | KRT7 | -1.156 | 0.009 |
| Observer | Syt1 | 0.957 | 0.009 |

| | | | |
|---|---|---|---|
| Observer | Ywhah | -2.127 | 0.012 |
| Observer | KRT6A | -1.431 | 0.016 |
| Observer | DCD | -1.190 | 0.016 |
| Observer | Eno1-ps1 | 2.012 | 0.019 |
| Observer | Dsg1 | -1.477 | 0.020 |
| Observer | Evpl | 2.231 | 0.022 |
| Observer | Fcgbp | -1.173 | 0.028 |
| Observer | KRT6C | -1.622 | 0.030 |
| Observer | Dsc1 | -1.201 | 0.030 |
| Observer | Tubb6 | -1.359 | 0.031 |
| Observer | KRT1 | -1.271 | 0.035 |
| Observer | Chtopl1 | 2.111 | 0.036 |
| Observer | KRT6B | -1.690 | 0.036 |
| Observer | Krt77 | -5.453 | 0.037 |
| Observer | Rab10 | 0.897 | 0.041 |
| Observer | KRT9 | -1.087 | 0.047 |
| Observer | Mylk2 | -1.245 | 0.048 |
| Observer | Krt73 | -1.593 | 0.049 |
| Pseudo | Evpl | 2.639 | 0.000 |
| Pseudo | Epb41l3 | -2.032 | 0.001 |
| Pseudo | Dpysl2 | -1.804 | 0.002 |
| Pseudo | Slc25a3 | 6.705 | 0.004 |
| Pseudo | Stxbp1 | -1.395 | 0.006 |

| | | | |
|---|---|---|---|
| Pseudo | Ka11 | -3.235 | 0.007 |
| Pseudo | Dcd | -1.045 | 0.009 |
| Pseudo | H1f2 | 1.873 | 0.010 |
| Pseudo | Tgm3 | -1.328 | 0.012 |
| Pseudo | Dsg1 | -1.282 | 0.014 |
| Pseudo | LOC102550284 | 0.893 | 0.016 |
| Pseudo | Cltc | 1.095 | 0.017 |
| Pseudo | Ywhah | -1.630 | 0.018 |
| Pseudo | Alyref | 0.968 | 0.020 |
| Pseudo | Syngr3 | 7.121 | 0.021 |
| Pseudo | Mylk2 | -1.448 | 0.022 |
| Pseudo | Tubb3 | 5.287 | 0.025 |
| Pseudo | Ewsr1 | 1.518 | 0.026 |
| Pseudo | Krt77 | -5.750 | 0.036 |
| Pseudo | Rps16 | -1.282 | 0.036 |
| Pseudo | Atp5po | -1.144 | 0.038 |
| Pseudo | LOC100361067 | -0.942 | 0.042 |

**Supplemental Table 4: K48 polyubiquitin targets in the ACC of male rats**

| **Group** | **Protein** | **log 2 FC** | ***p*-value** |
|---|---|---|---|
| Demonstrator | Dnm1 | 0.926 | 0.000 |
| Demonstrator | Fus | 1.645 | 0.000 |
| Demonstrator | Tubb3 | 1.404 | 0.001 |
| Demonstrator | Tubb4b | 1.077 | 0.001 |
| Demonstrator | Cltc | 0.784 | 0.001 |
| Demonstrator | Mdh1 | 1.324 | 0.001 |
| Demonstrator | Rbmx | 1.103 | 0.001 |
| Demonstrator | Ap2b1 | 1.319 | 0.002 |
| Demonstrator | Alyref | 1.162 | 0.002 |
| Demonstrator | Map6 | 1.423 | 0.003 |
| Demonstrator | Tubb2a | 1.095 | 0.003 |
| Demonstrator | Dpysl2 | 1.251 | 0.003 |
| Demonstrator | Atp5f1b | -3.059 | 0.004 |
| Demonstrator | Ap2a | 1.655 | 0.005 |
| Demonstrator | Nsf | 1.025 | 0.005 |
| Demonstrator | Gapdh | 1.005 | 0.005 |
| Demonstrator | Aldoa | 1.314 | 0.005 |
| Demonstrator | Actbl2 | 1.304 | 0.006 |
| Demonstrator | Crmp1 | 1.352 | 0.006 |
| Demonstrator | Caprin1 | 1.433 | 0.006 |

| | | | |
|---|---|---|---|
| Demonstrator | Rps25; RPS25 | 1.644 | 0.006 |
| Demonstrator | Tuba1a | 0.799 | 0.006 |
| Demonstrator | Ubc | 1.276 | 0.007 |
| Demonstrator | Arpc1a | 1.084 | 0.007 |
| Demonstrator | LOC687780 | 1.755 | 0.009 |
| Demonstrator | Map1a | 1.191 | 0.011 |
| Demonstrator | Pgk1 | 1.360 | 0.012 |
| Demonstrator | Dync1h1 | 1.000 | 0.012 |
| Demonstrator | Atp6v0a1 | 0.778 | 0.012 |
| Demonstrator | Ppia | 1.228 | 0.012 |
| Demonstrator | Tppp | 1.551 | 0.012 |
| Demonstrator | Ldhb | 1.310 | 0.013 |
| Demonstrator | Fscn1 | 1.118 | 0.014 |
| Demonstrator | Hnrnpk | 1.306 | 0.014 |
| Demonstrator | Tubb4a | 1.339 | 0.014 |
| Demonstrator | Carmil2 | 1.550 | 0.015 |
| Demonstrator | Rps7 | 1.291 | 0.016 |
| Demonstrator | Hnrnpa1 | 2.096 | 0.017 |
| Demonstrator | Cirbp | 0.998 | 0.018 |
| Demonstrator | Hsp90aa1 | 0.842 | 0.018 |
| Demonstrator | Pygb | 1.215 | 0.019 |
| Demonstrator | Got1 | 1.089 | 0.020 |
| Demonstrator | Kplce | -1.154 | 0.021 |

| | | | |
|---|---|---|---|
| Demonstrator | Dnm1 | 1.635 | 0.021 |
| Demonstrator | Pkm | 0.562 | 0.023 |
| Demonstrator | Got2 | 1.131 | 0.025 |
| Demonstrator | LOC497861 | 1.996 | 0.025 |
| Demonstrator | Ldha | 1.149 | 0.034 |
| Demonstrator | Wasf1 | 1.490 | 0.035 |
| Demonstrator | Syn1 | 1.054 | 0.036 |
| Demonstrator | Atp6v1h | 1.069 | 0.037 |
| Demonstrator | Gdi1 | 1.221 | 0.039 |
| Demonstrator | Cfl1 | 0.756 | 0.044 |
| Demonstrator | Mbp | 0.842 | 0.046 |
| Demonstrator | Atp6v1e1 | 1.478 | 0.049 |
| Pseudo | Ckmt1 | -3.566 | 0.001 |
| Pseudo | LOC100362366 | -1.890 | 0.010 |
| Pseudo | Ewsr1 | 1.371 | 0.021 |
| Pseudo | Pygb | 2.213 | 0.026 |
| Pseudo | Got1 | 1.499 | 0.031 |
| Pseudo | Caprin1 | 3.413 | 0.031 |
| Pseudo | Dsg | 1.790 | 0.040 |
| Pseudo | Atp5f1b | -1.579 | 0.044 |
| Observer | Rps25; RPS25 | 1.256 | 0.004 |
| Observer | Carmil1 | 2.092 | 0.004 |
| Observer | Map6 | 1.176 | 0.008 |

| | | | |
|---|---|---|---|
| Observer | Carmil2 | 1.109 | 0.009 |
| Observer | LOC100360413 | 0.938 | 0.017 |
| Observer | Wasf1 | 1.045 | 0.019 |
| Observer | Slc17a7 | 1.261 | 0.020 |
| Observer | Atp6v1e1 | 1.380 | 0.021 |
| Observer | Dpysl2 | 0.922 | 0.021 |
| Observer | Tubb4a | 1.028 | 0.031 |
| Observer | Rpl12-ps1 | 0.648 | 0.033 |
| Observer | Syn1 | 0.765 | 0.039 |
| Observer | Rbmx | 0.633 | 0.042 |
| Observer | LOC497861 | 1.522 | 0.044 |
| Observer | Fus | 0.943 | 0.045 |
